# Dynamics of pro-inflammatory and anti-inflammatory cytokine release during zcute inflammation in chronic obstructive pulmonary disease: an *ex vivo* study

**DOI:** 10.1186/1465-9921-9-47

**Published:** 2008-05-29

**Authors:** Tillie-Louise Hackett, Rebecca Holloway, Stephen T Holgate, Jane A Warner

**Affiliations:** 1 School of Biological Sciences, University of Southampton, Southampton, UK; 2 Infection, Inflammation and Repair Division, Southampton General hospital, Southampton, UK

## Abstract

**Background:**

Exacerbations of Chronic obstructive pulmonary disease (COPD) are an important cause of the morbidity and mortality associated with the disease. Strategies to reduce exacerbation frequency are thus urgently required and depend on an understanding of the inflammatory milieu associated with exacerbation episodes. Bacterial colonisation has been shown to be related to the degree of airflow obstruction and increased exacerbation frequency. The aim of this study was to asses the kinetics of cytokine release from COPD parenchymal explants using an *ex vivo *model of lipopolysaccharide (LPS) induced acute inflammation.

**Methods:**

Lung tissue from 24 patients classified by the GOLD guidelines (7F/17M, age 67.9 ± 2.0 yrs, FEV_1 _76.3 ± 3.5% of predicted) and 13 subjects with normal lung function (8F,5M, age 55.6 ± 4.1 yrs, FEV_1 _98.8 ± 4.1% of predicted) was stimulated with 100 ng/ml LPS alone or in combination with either neutralising TNFα or IL-10 antibodies and supernatant collected at 1,2,4,6,24, and 48 hr time points and analysed for IL-1β, IL-5, IL-6, CXCL8, IL-10 and TNFα using ELISA. Following culture, explants were embedded in glycol methacrylate and immunohistochemical staining was conducted to determine the cellular source of TNFα, and numbers of macrophages, neutrophils and mast cells.

**Results:**

In our study TNFα was the initial and predictive cytokine released followed by IL-6, CXCL8 and IL-10 in the cytokine cascade following LPS exposure. The cytokine cascade was inhibited by the neutralisation of the TNFα released in response to LPS and augmented by the neutralisation of the anti-inflammatory cytokine IL-10. Immunohistochemical analysis indicated that TNFα was predominantly expressed in macrophages and mast cells. When patients were stratified by GOLD status, GOLD I (n = 11) and II (n = 13) individuals had an exaggerated TNFα responses but lacked a robust IL-10 response compared to patients with normal lung function (n = 13).

**Conclusion:**

We report on a reliable *ex vitro *model for the investigation of acute lung inflammation and its resolution using lung parenchymal explants from COPD patients. We propose that differences in the production of both TNFα and IL-10 in COPD lung tissue following exposure to bacterial LPS may have important biological implications for both episodes of exacerbation, disease progression and amelioration.

## Background

Chronic obstructive pulmonary disease (COPD) is a major cause of mortality world wide and is predicted to be the third-leading cause of death by 2020[1]. COPD is defined by the American Thoracic society as a disease process involving progressive chronic airflow obstruction because of chronic bronchitis, emphysema or both[2]. Both the emphysematous destruction of lung tissue and the enlargement of air spaces along with excessive cough and sputum productions associated with bronchitis are believed to be related to an exaggerated inflammatory response[3]. Indeed the activation and infiltration of inflammatory cells including (CD8+) T lymphocytes, macrophages and neutrophils is a prominent feature of COPD[4,5]. In addition to the chronic state of inflammation observed in the airway patients with COPD are also prone to periods of exacerbation of the disease which are an important cause of the morbidity and mortality found in COPD [6-8]. COPD exacerbations are caused by a variety of factors such as viruses, bacteria and common pollutants. COPD exacerbations are now being recognised as important features of the natural history of COPD, as the frequency of exacerbations is associated with the severity of disease[9,10]. Statergies to reduce exacerbation frequency are thus urgently required and depend on an understanding of the inflammatory milieu associated with exacerbation episodes. The precise role of bacteria in COPD exacerbation has been difficult to asses due to approximately 30% of stable state COPD patients having bacterial colonisation within the airways[11]. The most common organism isolated from COPD patients is *Haemophilus Influenzae *and others include *streptococcus pheumoniae *and *Bramhemella carrarhalis*[11]. Bacterial colonisation has been shown to be related to the degree of airflow obstruction and increased exacerbation frequency[9,12-14]. More recently Stockley and colleagues have shown that COPD exacerbations associated with purulent sputum are more likely to produce positive bacterial cultures than exacerbations where the sputum was mucoid[15]. Additionally Sethi and collegues have shown that exacerbations associated with H. *influenza *and B. *catarrhalis *both gram negative bacteria are associated with significantly higher levels of inflammatory markers compared to pathogen-negative exacerbations[16].

Wedzicha and colleagues have shown that stable state COPD patients with high sputum levels of Interleukin-6 (IL-6) and CXCL8 have more numerous exacerbations, suggesting that the frequency of exacerbations is associated with increased airway inflammation[17,18]. Cytokines such as IL-6 and CXCL8 are rarely produced individually instead they are more usually released in combination with other cytokines and mediators that are characteristic of a particular disease state. These cytokine networks exhibit great pleiotropy and redundancy to the effect that any one cytokine may be influenced by another released simultaneously. TNFα and IL-1β have been identified as key cytokines that are able to initiate inflammatory cascades during exacerbations of chronic inflammatory conditions such as rheumatoid arthritis, inflammatory bowel disease, and severe asthma [19-21]. Although it is presumed that COPD exacerbations are associated with increased airway inflammation, as in patients with asthma, there is little information on the nature of the inflammatory mediator milieu during an exacerbation, especially when studied from the onset of symptoms.

In this study we aimed to assess the kinetics of key pro- and anti-inflammatory cytokines released from lung parenchymal explants obtained from COPD patients, using an *ex vivo *model of Gram negative Lipopolysaccharide (LPS) induced acute inflammation. We found that COPD disease severity was associated with an enhanced *ex vivo *pro-inflammatory cytokine response led by TNFα which was not ameliorated by the anti-inflammatory cytokine IL-10.

## Methods

### Patient characteristics for human lung tissue experiments

Human parenchymal lung tissue was obtained from 37 patients (15F/21M) undergoing resection for carcinoma and 1 male undergoing surgery to remove a cyst at Guy's Hospital, London. All specimens of parenchymal tissue were obtained from sites distant from the tumour. The study was approved by the institutional ethics committee and all volunteers gave informed consent. The Global Initiative for Chronic Obstructive Pulmonary Disease (GOLD) uses a four step classification for the severity of COPD based on measurements of airflow limitation during forced expiration[22,23]. Each stage is determined by the volume of air that can be forcibly exhaled in one second (FEV_1_) and by the ratio of FEV_1 _to the forced vital capacity (FVC); lower stages indicate less severe disease. Using the GOLD guidelines our patient cohort was stratified into the following groups, GOLD I (FEV_1_/FVC < 70%, FEV_1 _≥ 80% predicted), GOLD II (FEV_1_/FVC < 70%, 50% ≤ FEV_1 _< 80% predicted) and individuals with normal lung function (FEV_1_/FVC > 70%, FEV_1 _≥ 90% predicted)[23]. Table 1 shows the number of patients in each GOLD stage and their demographics which include age, gender, lung function and smoking history. For the purposes of this study ex-smokers were defined as individuals that had given up smoking for ≥ 3 years to ensure for smoking cessation. All demography data was available up to the date of surgery and none of the subjects were treated prior with inhaled or oral corticosteroids or bronchodilators.

**Table 1:** Patient characteristics of subjects prior to the removal of lung tissue

**Classification**	**Normal Lung Function**	**GOLD I**	**GOLD II**
	FEV_1_/FVC > 70% FEV_1 _≥ 90% predicted	FEV_1_/FVC < 70% FEV_1 _≥ 80% predicted	FEV_1_/FVC < 70% 50% ≤ FEV_1 _< 80% predicted
**No. subjects**	13	11	13
**Age**	55.6 ± 4.1	69.2 ± 2.9	66.9 ± 2.8
**Gender**	8 F 5 M	3 F 8 M	4 F 9 M
**Lung function (FEV_1_/FVC)**	0.82 ± 0.02	0.63 ± 0.03	0.59 ± 0.02
**FEV_1 _% predicted**	98.8 ± 4.1	90 ± 4.0	65.6 ± 2.4
**Smoking status**	6 current smokers	6 current smokers	8 current smokers
	4 ex-smokers	5 ex-smokers	5 ex smokers
	3 non-smokers		

Tissue samples were taken from 37 patients. Patient details including age, gender, lung function given as the ratio of air that can be forcibly exhaled in one second (FEV_1_) to the forced vital capacity (FVC), FEV_1_/FVC and FEV_1_% predicted and smoking status are listed as the mean ± SEM.

### Preparation of human lung tissue for primary cell culture

The procedure for preparation of human lung tissue has been described previously elsewhere[24]. Briefly, resected lung tissue was dissected free of tumour, large airways, pleura and visible blood vessels and finely chopped using dissecting scissors, into 2 mm^3 ^fragments during several washes with Tyrode's buffer containing 0.1% sodium bicarbonate. Six explants (total weight approx. 30 mg) were incubated per well (2.0 cm^2^) of a 24 well plate with RPMI-1640 medium containing 1% penicillin, 1% streptomycin, and 1% gentamycin at 37°C in 5% carbon dioxide/air for 16 hours. Tissue was then either stimulated with 100 ng/ml LPS (Sigma-Aldrich, UK) or maintained in cell culture media alone for 1, 2, 4, 6, 24, or 48 hours. For neutralisation of TNFα and IL-10 bioactivity, tissue was incubated with 1 μg/ml of neutralising TNFα or IL-10 antibody or an isotype control (R&D Systems, Minneapolis, USA) for 1 hr prior to stimulation with 100 ng/ml LPS. Lung tissue fragments and supernatant were harvested at each time point and both were stored at -80°C until analysis. The tissue fragments were weighed to determine total tissue weight to normalize the levels of released cytokines.

### Immunohistochemistry of human lung tissue

For the last 18 individuals recruited in the study the lung explants collected (6 per experimental condition) were embedded in glycol methacrylate (GMA), following stimulation with LPS or cell culture media alone for 1 or 6 hrs, as described above. The patient demographics which include age, gender, lung function, GOLD stage and smoking history as well as the mean number of macrophages, mast cells and lymphocytes counted for each group determined by lung function are given in table 2. To determine the cell types responsible for TNFα release in response to LPS, immunohistochemical staining of the samples was conducted as previously described[25]. Briefly, serial sections of 2 μM were stained immunohistochemically using the streptavidin biotin-peroxidase detection system and murine monoclonal antibodies directed to either human TNFα (1:100, clone 2B3A6A2, Biosource, SA), CD68 (1:200, clone PG-M1, DAKO), mast cell tryptase (1:1000, clone AA1, DAKO) or neutrophil elastase (1:500, clone NP57, DAKO). Control sections were incubated with isotype-matched immunoglobulins. The previously described camera-lucida technique was used to determine which cells per mm^2 ^of alveolar tissue co-localised with TNFα positive staining on the serial sections[26].

**Table 2:** Numbers of Macrophages, Mast cells and Neutrophils in lung tissue from COPD patients and individuals with normal lung function

	**Normal lung Function (4M/6F)**	**GOLD I/II (6M/4F)**	**p Value**
**Lung function (FEV_1_/FVC)**	0.79 ± 0.02	0.62 ± 0.03	0.05
**FEV_1 _% predicted**	99.2 ± 9.7	77.2 ± 8.5	0.05
**Smoking status**	4 current smokers	4 current smokers	
	3 ex-smokers	5 ex-smokers	
	2 non-smokers		
**Age**	64.7. ± 7.9	71.2 ± 2.0	0.07
**Macrophage (CD68) cell/mm**^2^	2.8 ± 0.6	5.4 ± 1.4	0.10
**Mast Cell (Tryptase) cell/mm**^2^	20.6 ± 5.5	17.1 ± 3.9	0.23
**Neutrophil (Neutrophil elastase) cell/mm**^2^	8.1 ± 0.7	11.5 ± 3.6	0.15

Patient data including lung function given as the ratio of air that can be forcibly exhaled in one second (FEV_1_) to the forced vital capacity (FVC), FEV_1_/FVC and FEV_1_% predicted, smoking status, age and gender are listed as the mean ± SEM. Cell numbers are listed as the mean number of cells/mm^2^, ± SEM and the p value obtained when comparing the each factor between the two groups is given, p < 0.05 was considered statistically significant.

### Enzyme-Linked Immunosorbent Assay

The levels of each cytokine in the supernatant were measured by enzyme-linked immunosorbent assay (ELISA) and the concentration corrected for tissue weight. Human TNFα and IL-1β specific ELISA kits (limit of detection of 0.3 pg/mg of tissue and 0.1 pg/mg of tissue, respectively) were purchased from R&D Systems Europe Ltd, Abingdon, UK. Human IL-5, IL-6, CXCL8 and IL-10 were all measured using commercially available ELISA Duosets from Biosource Europe, SA (limits of detection 0.3 pg/mg of tissue, 0.28 pg/mg of tissue, 0.26 pg/mg of tissue and 0.25 pg/mg of tissue, respectively). The manufacturer's protocol was followed for each ELISA.

### Lactate dehydrogenase assay

To test for tissue viability Lactate dehydrogenase (LDH) levels were measured in lung supernatant using a commercially available assay and LDH standard from Roche (Indianapolis, USA). For a positive control, lung explants were homogenised on ice using a XL10 sonicator set at an amplitude of 2 microns, for 12 cycles of 10 seconds sonication followed by 20 seconds rest, in 10% triton PBS buffer containing protease inhibitor cocktail (P2714, Sigma-Aldrich, UK). Following sonication samples were centrifuged at 15,000 g for 15 mins at 4°C, and supernatant removed for storage. The limit of detection for the assay was 1.95 ng/mg of tissue.

### Statistical Analysis

All results were normalised using the tissue weight and are expressed as the mean ± SEM. Before statistical evaluation, all results were tested for population normality and homogeneity of variance, and where applicable, a Student *t *test was performed. A value of P < 0.05 was accepted as significant. Differences within standard curves were analysed by ANOVA with a Tukey/Kramer post hoc correction again a value of P < 0.05 was accepted as significant. Correlations between parameters were examined for statistical significance by Spearman's correlation. Experiments were performed on each of the patients in the cohort.

## Results

### Kinetics of the acute inflammatory response in human lung tissue

**Figure 1:**
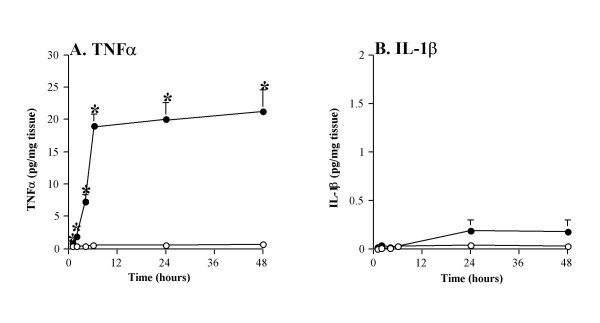
**Kinetics of the acute inflammatory response in human lung tissue**. Human lung tissue (n = 37) was stimulated with 100 ng/ml LPS (filled circles) or buffer control (open circles). The release of (A) TNFα and (B) IL-1β into the supernatant was measured by commercial ELISA. Values shown are the mean ± SEM and are expressed as pg/mg of tissue, * indicates a p value < 0.05.

Release of the pro-inflammatory cytokine TNFα was significantly higher in the LPS stimulated tissue as early as 1 hr, continued to rise at 2 and 4 hrs, and peaked at 6 hrs (mean = 17.4 ± 1.5 pg/mg of tissue) compared to undetectable levels in the non-stimulated controls (Figure 1A). Release of TNFα from LPS-stimulated tissue was dose-dependant within the range of 0.1–1000 ng/ml, with a maximal response at 1000 ng/ml therefore, in subsequent experiments, we used a sub-maximal LPS dose of 100 ng/ml. Over a 48 hr time period there was no change in the levels of LDH in supernatants from LPS stimulated tissue, compared to buffer, indicating the absence of cytotoxic effects. While LPS can potentially activate a range of different cell types, not all pro-inflammatory cytokines were released. Figure 1B shows that when the tissue was stimulated with LPS or buffer for 48 hrs there was no statistical significant difference in the levels of IL-1β released.

### Cytokine cascades in the acute inflammatory response

**Figure 2:**
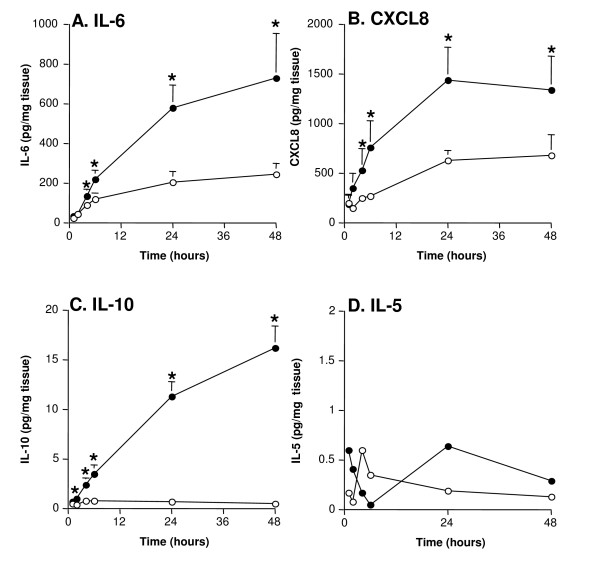
**Cytokine cascades in the acute inflammatory response**. Human lung tissue (n = 37) was stimulated with 100 ng/ml LPS (filled circles) or buffer control (open circles). The supernatants were analysed for (2A) IL-6, (2B) CXCL8, (2C) IL-10 and (2D) IL-5 using commercially available ELISAs. For all values are the mean ± SEM and are expressed as pg/mg tissue. * indicates p < 0.05.

As shown in figure 2A the maximal increase of IL-6 occurred later than TNFα, peaking at 48 hrs (mean = 685.7 ± 189 pg/mg of tissue) compared to tissue challenged with buffer alone (mean = 238.3 ± 50 pg/mg of tissue, P < 0.05). The release of the chemokine CXCL8 followed a similar pattern to IL-6, with a maximal response occurring at 24 hrs (mean = 1490.4 ± 394 pg/mg of tissue) versus tissue challenged with buffer (mean = 692.3 ± 251 pg/mg of tissue, P < 0.05) (figure 2B). The levels of anti-inflammatory cytokine IL-10 were still increasing between 24 hrs and 48 hrs (mean = 15.2 ± 2.4 pg/mg of tissue) compared to undetectable levels in the tissue challenged with buffer (P < 0.05, figure 2C). In contrast, IL-5 was not released in response to LPS (figure 2D).

### TNFα release at 6 hours predicts subsequent cytokine levels at 24 hours

**Figure 3:**
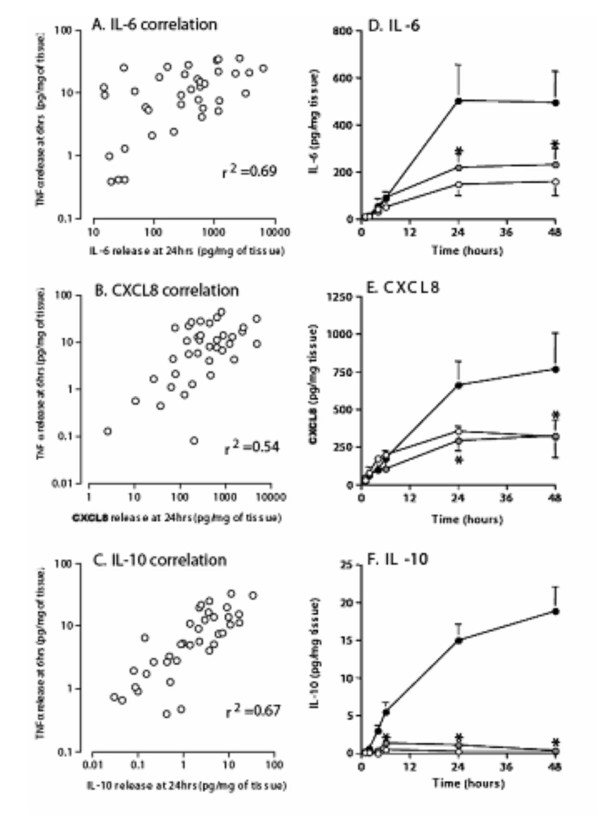
**TNFα, the key cytokine in the inflammatory response**. Data from figures 1A and 2A, B, and 2C were re-plotted to analyse the relationship between TNFα release at 6 hrs and IL-6 (3A), CXCL8 (3B) and IL-10 (3C) release at 24 hrs. Data was analysed using Spearman rank correlation, the values given are the Rho and p < 0.05. To confirm the role of TNFα in the cytokine cascade human lung tissue (n = 37) was pre-treated with neutralising TNFα antibody (nTNFαAb) (grey circles) or an isotype control (open circles) for 1 hr and then stimulated with 100 ng/ml LPS (filled circles). The supernatants were analysed for IL-6 (3D), CXCL8 (3E), and IL-10 (3F) using commercial ELISAs. For all values given are the mean ± SEM and are expressed as pg/mg of tissue * indicates a P value < 0.05.

The kinetic data indicated that a succession of cytokines are released in response to LPS, with TNFα reaching maximal release first. We examined the relationship between the levels of TNFα released at 6 hrs and the levels of the other cytokines measured at 24 hrs (Figures 3A, B and 3C). The resultant data indicated that the amount of TNFα released at 6 hrs could be used to predict IL-6, CXCL8 and IL-10 release at 24 hrs.

If TNFα is a key initiating step in the inflammatory cascade, then removal of TNFα should arrest or attenuate subsequent cytokine release. Pre-treatment of explants with a TNFα neutralising antibody (nTNFα Ab) for 1 hr before LPS stimulation reduced the release of IL-6 and CXCL8 back to baseline levels and the effect was still evident at 48 hrs post stimulation compared to treatment with an isotype control and LPS (figures 3D &3E). Pre-treatment with nTNFα Ab also completely abrogated the release of IL-10 up to 48 hrs after LPS stimulation (figure 3F).

### Co-localisation of TNFα with macrophages and mast cells in response to LPS

**Figure 4:**
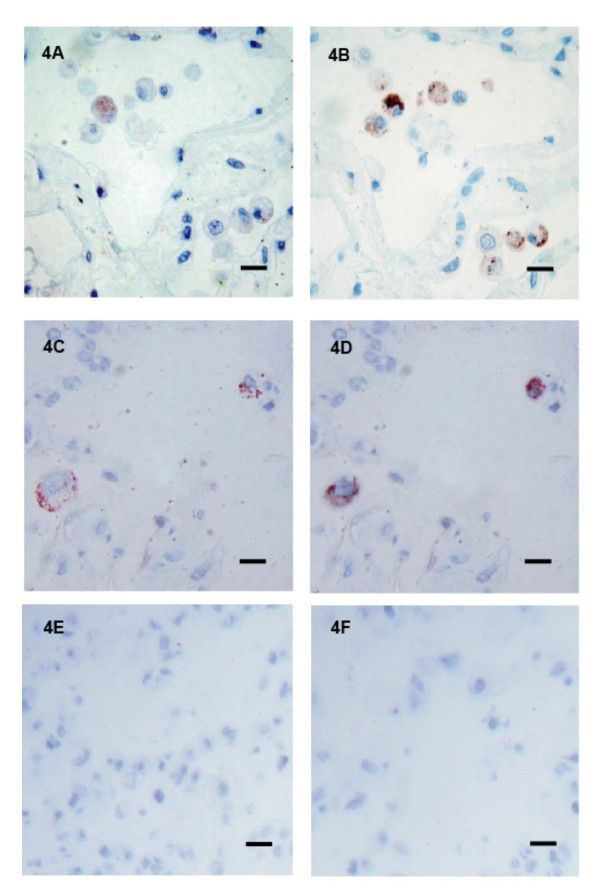
**Co-localisation of TNFα with macrophages in the lung parenchyma**. Lung tissue was obtained from a 65 year-old female smoker (GOLD 1) stimulated with LPS for 1 hour. The tissue was then embedded and sequential sections of the lung parenchyma stained with monoclonal antibodies for TNFα (figure 4A and 4C) and CD68 (4B) and mast cell tryptase (4D). Staining specificity was determined by IgG_1 _isotype antibody controls 1:200 (4E) and 1:1000 (4F) for CD68 and mast cell tryptase respectively. Bars represents 10 μm, positive cells are stained red.

As we demonstrated in figure 1A that the release of TNFα was statistically elevated after 1 hour of LPS exposure it was important to determine which cell/cells were responsible for this early TNFα release. The cellular source of TNFα was analysed in 18 subjects (9F/9M) of the study consisting of 8 current, 7 ex and 3 non-smokers with a range of lung functions (FEV_1 _% predicted 55 – 92%). To determine the inflammatory cell types responsible for TNFα release, serial sections were stained for TNFα and one of the following cell markers: neutrophil elastase (neutrophils), CD68 (macrophages), or mast cell tryptase (mast cells). All sections stained positively for varying amounts of neutrophil elastase, CD68 and mast cell tryptase. Figure 4 shows a representative section of lung parenchyma from a 65-year-old female smoker (FEV_1 _83% predicted), immuno-stained with anti-TNFα monoclonal antibody after 1 hr exposure to LPS (see figure 4A and 4C) and the serial sections stained for CD68 (see figure 4B) and mast cell tryptase (see figure 4D). Co-localisation of TNFα occurred in association with macrophages and mast cells after 1 hr of exposure to LPS, and was consistent for all individuals studied. TNFα did not co-localise with neutrophil elastase staining. We also analysed tissue following 6 hours of LPS exposure however we found no difference in the cellular sources of TNF alpha. As shown in table 2, within the parenchymal tissue collected we found no statistically significant differences in the numbers and distribution of macrophages, mast cells or neutrophils within the tissue obtained from GOLDI/II patients compared to individuals with normal lung function.

### IL-10, a negative regulator of TNFα production

**Figure 5:**
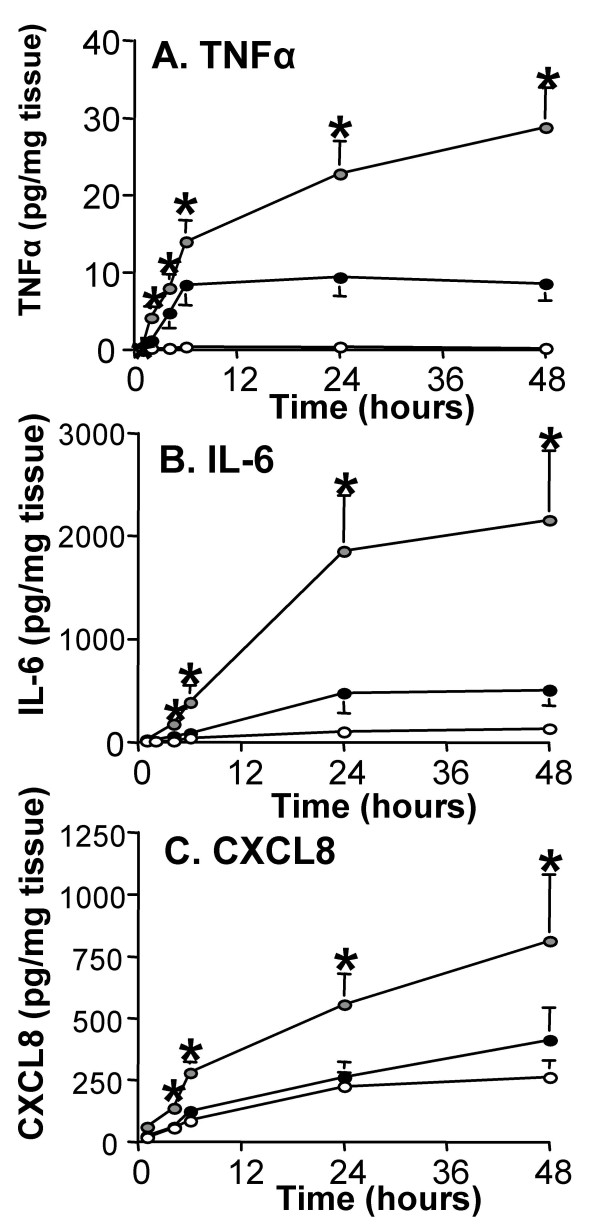
**IL-10, a negative regulator of TNFα production**. Human lung tissue (n = 37) was pre-treated with neutralising IL-10 antibody (nIL-10Ab) (grey circles) or an isotype control (open circles) for 1 hr and then stimulated with 100 ng/ml LPS (filled circles). The supernatants were analysed for (A) TNFα, (B) IL-6, and (C) CXCL8 using commercially available ELISAs. Values given are the mean ± SEM and are expressed as pg/mg of tissue, * indicates a P value < 0.05.

IL-10 has been shown to act as a negative regulator of TNFα production [27,28]. We were therefore interested in studying the effects of IL-10 and whether it was able to regulate the release of TNFα. Pre-treatment with an IL-10 neutralising antibody (nIL-10Ab) for 1 hour before LPS stimulation augmented the release of TNFα (figure 5A), particularly at the later time points where we previously observed maximal IL-10 release (figure 2C). Since neutralising the activity of IL-10 resulted in augmented release of TNFα, we next examined if there was a similar increase in the release of any other cytokines involved in the inflammatory cascade. Pre-treatment with nIL-10 Ab also resulted in a significantly augmented release of both IL-6 and CXCL8 at 24 hrs, which was maintained for at least 48 hrs (figures 5B &5C).

### Severity of COPD influences cytokine release

**Figure 6:**
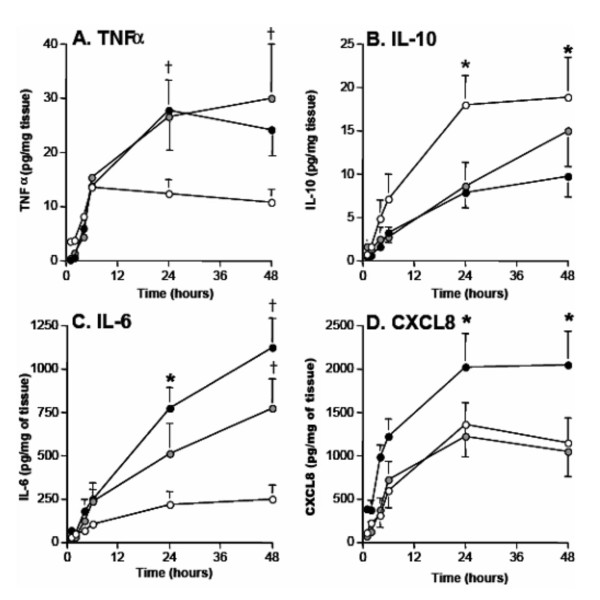
**Severity of COPD influences cytokine release**. Using the GOLD guidelines the 37 individuals in this study were classified as GOLD I (grey circles) and GOLD II (filled circles) or subjects with normal lung function (open circles). Data from figures 1A, 2A, 2B and 2C were then re-analysed to determine the kinetics of (A) TNFα, (B) IL-10, (C) IL-6 and (D) CXCL8 release from the lung tissue of the patients in the three classified groups. Values given are the mean ± SEM and are expressed as pg/mg of tissue, † indicates P < 0.05 for both GOLD I and GOLD II compared to GOLD 0, and * indicates P < 0.05 for GOLD II compared to GOLD 0.

We observed large variation in cytokine release between individuals and therefore sought to assess if there was an association between lung function and cytokine release in our explant model. Patients were classified into the following groups, normal lung function (n = 13), GOLD I (n = 11) and GOLD II (n = 13) using the GOLD guidelines[23]. We observed that all patients showed a similar level of TNFα release up to the 6 hr time point, however at 24 hrs, TNFα release continued to increase in individuals classified as GOLD I and GOLD II (mean = 24.7 ± 3.3 and 27.6 ± 4.2 pg/mg of tissue respectively), when compared to patients with normal lung function (mean = 13.7 ± 2.1 pg/ml of tissue, P < 0.05; figure 6A). By 48 hrs TNFα release plateaued in all groups. Release of IL-6 and CXCL8 followed a similar pattern to that observed for TNFα with GOLD II explants releasing elevated levels of these mediators at 24 and 48 hrs (figures 6C and 6D). In contrast, IL-10 release from GOLD I (mean = 8.5 ± 2.7 pg/mg of tissue) and GOLD II patients (mean = 7.8 ± 1.8 pg/mg of tissue) was actually lower compared to patients with normal lung function (mean = 17.9 ± 3.1 pg/mg of tissue, P < 0.05) (see figure 6B). Importantly for all of the patient demographic data collected including age, gender, and smoking status these data did not influence cytokine release in response to LPS.

## Discussion

In this study, we have employed an *ex vivo *lung explant model to investigate the initial acute inflammatory response initiated by exposure to Gram negative bacterial cell wall component LPS in lung tissue derived from COPD patients and normal individuals. We demonstrate that lung explants obtained from COPD patients classified with mild to moderate airflow obstruction (GOLD I and II) release elevated concentrations of pro-inflammatory cytokines TNFα, IL-6 and CXCL8 in response to LPS but failed to mount an appropriate anti-inflammatory IL-10 response when compared to normal lung tissue. We suggest that these findings may have important clinical implications for the pathogenesis of COPD as dysregulated resolution of inflammation by IL-10 could account for the exaggerated inflammation observed in COPD patients during episodes of exacerbation.

The association between bacterial colonization and the development and progression of airway inflammation in COPD has been a subject of study for several years[29,30]. Although bacteria such as H. *influenzae *have been associated with COPD exacerbation, early studies have provided conflicting results as to its isolation during exacerbation [12-15]. Later evidence for the role of bacteria in COPD exacerbations has come from antibiotic therapy studies. Hill and colleagues in a large COPD study showed that the airway bacterial load was related to inflammatory markers and that the bacterial species present was related to the degree of inflammation[31]. Although the subsequent inflammatory response following a bacterial infection is considered to play a key role in the pathogenesis of COPD, the nature and sequence of the cytokine networks involved in an exacerbation have remained unexplored. The majority of clinical studies have previously concentrated on examining the acute inflammatory response during exacerbations of COPD patients using induced sputum and bronchial alveolar lavage (BAL) fluid. To our knowledge this is the first study to compare explants from patients with characterised COPD and individuals with normal lung function to investigate the kinetics of the acute inflammatory cytokine response within the distal lung towards LPS, a bacterial wall component. LPS is a widely used stimulus that acts on a number of cells within the lung through well-defined signalling cascades [32-34]. Within the literature the typical dose of LPS used in cell culture experiments and rodent models of airways disease is 1 μg/ml [35-37]. We carried out dose response curves for LPS on the tissue and deliberately chose a sub-maximal concentration of LPS 0.1 μg/ml in order to explore cytokine release and interactions on a number of cells within the lung explants.

In our model of acute inflammation in human lung tissue we found that TNFα, IL-6 and CXCL8 were released following stimulation with LPS. This model using LPS mimics the cytokine profile previously reported by several groups in COPD patients with bacterial infections. In particular Solar and colleagues showed that the presence of potentially pathogenic organisms in the bronchoaleolar lavage from COPD patients was associated with a greater degree of neutrophillia and higher TNFα levels[13]. Indeed several studies have confirmed that higher bacterial load is associated with greater airway inflammation measured by elevated TNFα, IL-6 and CXCL8 in BAL fluid from COPD patients[13,38]. Additionally several exacerbation studies have reported elevated levels of TNFα, IL-6 and CXCL8 in induced sputum from COPD patients admitted to hospital following an exacerbation[9,39]. Although bacterial load was not assessed in these exacerbation studies the cytokines reported, TNFα, IL-6 and CXCL8 are the same cytokines that we observe in our acute inflammatory model using LPS. The advantage of this model over *in vivo *studies is that we have been able to determine the kinetic profile of release of the cytokines most reportedly elevated in COPD patients during exacerbations.

Classification of the patients in our study using the GOLD guidelines for COPD diagnosis allowed us to segregate patients into those with normal lung function and those with mild (GOLD I) and moderate (GOLD II) COPD[23]. Using this approach, we found that lung explants from patients with GOLD I and II status had an elevated TNFα and subsequent IL-6 and CXCL8 response compared to explants obtained from patients with normal lung function. Our data therefore suggests that the parenchyma tissue of an individual with COPD would respond with an enhanced inflammatory response following exposure to LPS. The relationship between the magnitude of the inflammatory response and disease severity in our study may therefore have important clinical implications. Recent findings indicate that some patients with COPD develop frequent exacerbations, and recurrent exacerbations may be associated with increased airway inflammation. Indeed Bhowmik *et al*.,[17] reported that COPD patients with elevated concentrations of IL-6 and CXCL8 in sputum were more likely to have frequent exacerbations, which is thought to lead to the rapid decline of lung function in these patients. In support of these findings other studies have also demonstrated a negative correlation between FEV_1 _and the levels of TNFα, IL-6 and CXCL8 in sputum[39] and BAL fluid[13,38]. These *in vivo *studies therefore provide biological significance to our findings that release of TNFα, IL-6 and CXCL8 from explants *in vitro *negatively correlates with patients lung function. Altogether the data suggests that the heightened inflammatory response in both our model and *in vivo *studies of exacerbations may lead to the accelerated decline in lung function observed in COPD patients and therefore has prognostic importance for the disease. In support of these finding Donaldson and colleagues have previous reported that exacerbations in moderate to severe COPD patients contribute a greater extent to the accelerated decline in FEV_1 _per year observed in these patients[40]. In addition to the role of exacerbations in COPD progression the work of Hurst and colleagues has recently raised important awareness to the impact exacerbations have on systemic inflammation as they have shown that the degree of systemic inflammation observed in COPD patients is related to the extent of lower airway inflammation during exacerbation[41]. These data bring focus to the accumulating evidence of extra pulmonary manifestations in COPD including cachexia and systemic inflammation which are observed in severe COPD patients. In our model of acute inflammation we observed with disease severity elevated release of cytokines such as IL-6 which could act systemically on the liver to promote fibrinogen production. As raised levels of plasma fibrinogen is a independent risk factor of for cardiovascular disease[42]. Future studies using whole animal models would therefore be useful to determine the role exacerbation derived inflammatory mediators play in systemic inflammation

In our study TNFα was the initial and predictive cytokine released in the cascade following LPS exposure. Given the heterogeneity of lung tissue obtained it was of interest to characterize which cells were responsible for the TNFα release in our model. Applying immunohistochemistry to GMA sections, we found that macrophages and mast cells accounted for the majority of TNFα positive cells following LPS exposure. This finding is supported by previous data showing that endotoxins of both Gram positive and Gram negative bacteria stimulate TNFα release from both these cell types[26,27]. Although we observed a 0.92 fold increase in the number and distribution of TNFα positive cells between GOLD I/II patients and controls this difference did not reach statistical significance. An extensive small airway study by Hogg et al[43] has previously reported that the percentage of airways positive for macrophages and neutrophils is elevated in the moderate to severe stages of COPD. It is difficult to compare our observations due to the differences in atomical location of the tissue analysed, small airways verses parenchyma and the methodologies used and additonally mast cells were not analysed in the Hogg *et al *study. Our investigations have only been able to focus on a narrow window of the disease spectrum due to the nature of patients undergoing surgery and therefore we are unable to include GOLD III and IV patients. Therefore it is difficult to determine if the increase in the numbers of macrophages with increasing COPD severity is responsible for the elevated levels of TNFα observed or that macrophages and mast cells in COPD patients have an exaggerated TNFα response due to pre-sensitisation. Indeed it has been shown that pre-sensitisation with LPS promotes an exaggerated Th_1 _cytokine response in mouse models of allergic asthma[44]. Future studies are therefore required to determine if pre-sensitisation of lung tissue to bacterial agents is related to the degree of inflammation observed in COPD patients[31].

If TNFα is a key cytokine in acute airway inflammation then neutralising its biological activity could provide an important therapeutic treatment if given early enough after a COPD exacerbation. Indeed, in our model inhibition of TNFα activity prevented the release of IL-6, CXCL8 and IL-10 following LPS exposure. Blockade of TNFα activity using monoclonal antibodies or the soluble TNFα receptor has been used as an effective therapy in rheumatoid arthritis, inflammatory bowel disease and severe asthma [19-21]. However published reports of two clinical trials which examined the effects of the chimeric monoclonal TNFα antibody infliximab (Remicade) in COPD patients found no improvement in symptoms, lung function or reduction of inflammation in induced sputum[45,46]. The failure of anti-TNFα therapies may reflect the fact that COPD is a highly complex inflammatory disease in which many mediators are involved. However, the substantial increase in TNFα production following LPS exposure in our model and *in vivo *exacerbation studies suggests that the role of TNFα may be more predominant in acute inflammatory episodes rather than in the chronic disease process. Therefore future studies maybe better focused on the roles of anti-TNF therapies in preventing or modifying the severity of acute exacerbations.

Several studies have shown that IL-10 acts as a classical negative feedback inhibitor on TNFα release from macrophages[27,47]. In support of this mechanism of action, we report that neutralization of IL-10 activity significantly augmented LPS stimulated TNFα release from lung explants. Release of IL-6 and CXCL8 were also shown to be augmented following IL-10 inhibition, although this was likely a direct result of the increased levels of TNFα. We also show that IL-10 release was completely abolished by neutralisation of the initial cytokine in the cascade, TNFα. This supports a role for a delicate cytokine balance between pro-inflammatory TNFα and anti-inflammatory IL-10 in both resolution of inflammation and normal homeostasis of the lung. Our finding that lung tissue from GOLD I and GOLD II COPD patients releases decreased levels of IL-10 in LPS derived acute inflammation compared to patients with normal lung function has potential important pathophysiologic relevance. In support of our finding Takanashi et al[48] have also reported evidence of IL-10 disregulation in COPD as they demonstrated that the level of IL-10 in sputum from COPD patients is decreased in comparison with healthy non-smokers. As decreased expression of the anti-inflammatory mediator IL-10 could lead to the enhanced TNFα released observed in the COPD explants in this study. This raises important questions as to the balance of pro and anti-inflammatory mediators released within the lung during exacerbations and their cause or effect relationship to the inflammatory profile observed in COPD. One possible mechanism for altered IL-10 gene expression could be single nucleotide polymorphisms (SNP) within the gene. To date no consensus has been reached regarding any IL-10 SNP in the progression of COPD. Alternatively IL-10 gene expression could be altered epigenetically due to environmental insults such as cigarette smoke or the oxidants released in response to smoke exposure. Future studies will hopefully provide more information as to the mechanisms and outcomes involved in these modifications and their role in disease progression. As therapeutic approaches aimed at preventing the inflammatory cascade in COPD are currently focused on pro-inflammatory mediators, anti-inflammatory interventions could therefore be equally if not more important. Since IL-10 is able to ameliorate the release of TNFα in acute inflammation, therapeutic strategies which enhance the endogenous release or activity of IL-10 could be used to dampen TNFα responses without compromising the immune system, providing important targets as new therapeutic strategies for a major clinical unmet need.

Due to the nature of COPD exacerbations it is technically difficult to investigate the kinetics of acute inflammatory events within the lung following admission of patients to hospital. In this *ex vivo *lung explant model, we have been able to interrogate further the acute inflammatory profile in terms of the tissue's response to LPS. The use of lung explants has several advantages over isolated cell cultures, including preservation of normal tissue architecture and cellular interactions. In addition, explants can be manipulated to dissect the role of various resident cells and specific cytokines they release using neutralizing antibodies. Using this model we have been able to clarify the intrinsic response of resident cells within the lung tissue following LPS exposure and eliminate the contribution of cytokine release from circulating cells. Therefore the model also has some disadvantages as it does not entirely mimic the *in vivo *situation as we have not studied the role of recruited inflammatory cells following LPS exposure. Another disadvantage is the fact that lung explants are extremely heterogenous between individuals especially COPD patients, and we have tried to account for this by selecting 6 explants randomly per experimental condition. Additionally all of the explants used were dissected free of small airways and therefore the model does not represent the contribution of small airways following LPS exposure. Other causes of COPD exacerbations include viruses and common pollutants; the role of bacterial-viral or bacterial-pollutant interactions may exist and have not been investigated in this study.

## Conclusion

In summary, we report on a reliable *ex vitro *model for the investigation of acute lung inflammation and its resolution using lung parenchymal explants from COPD patients. Using this model, we propose that differences in the production of both TNFα and IL-10 in COPD lung tissue following exposure to bacterial endotoxin LPS may have important biological implications for both episodes of exacerbation, disease progression and amelioration. Thus further work is required to determine the role of bacterial colonization, exacerbations and airway inflammation in the pathogenesis of COPD.

## Competing interests

Professor ST Holgate has received research funding from Celltech, Wyeth and Centercor in relation to the potential role of TNFa in severe asthma and has consulted with these 3 companies and UCB over the clinical trials of anti-TNF therapy in asthma

## Authors' contributions

TLH carried out the tissue culture studies, immunoassays, immunohistochemistry, performed the statistical analysis and drafted the manuscript, RH participated with the immmunohistochemistry, STH participated in the design of the study and helped draft the manuscript, JAW conceived of the study, participated in its design, coordination and helped draft the manuscript. All authors read and approved the final manuscript.
